# Supplementary material for: Diagnostic accuracy of vision-language models on Japanese diagnostic radiology, nuclear medicine, and interventional radiology specialty board examinations
Source: Jpn J Radiol. 2024 Jul 20;42(12):1392–8. doi: 10.1007/s11604-024-01633-0 (PMC11588758; doi:10.1007/s11604-024-01633-0)
Supplement: Supplementary file 1 — Supplementary file1 (PDF 31 KB) [file 11604_2024_1633_MOESM1_ESM.pdf]

Supplemental table 1 Accuracy rates of GPT4, GPT4V, and GPT4o-based ChatGPT, Claude3 Sonnet, and Claude3 Opus on the Japanese diagnostic radiology board certification test

	All questions					Question with images					Question without images					Single-answer question					Multi-answer question				
	GPT4 and 4V-ChatGPT	GPT4o-ChatGPT	Claude3 Sonnet	Claude3 Opus	No. of all questions	GPT4 and 4V-ChatGPT	GPT4o-ChatGPT	Claude3 Sonnet	Claude3 Opus	No. of question with images	GPT4 and 4V-ChatGPT	GPT4o-ChatGPT	Claude3 Sonnet	Claude3 Opus	No. of question without images	GPT4 and 4V-ChatGPT	GPT4o-ChatGPT	Claude3 Sonnet	Claude3 Opus	No. of single-answer question	GPT4 and 4V-ChatGPT	GPT4o-ChatGPT	Claude3 Sonnet	Claude3 Opus	No. of multi-answer question
2019	29 (36%)	38 (48%)	23 (29%)	33 (41%)	80	27 (36%)	36 (48%)	22 (29%)	30 (40%)	75	2 (40%)	2 (40%)	1 (20%)	3 (60%)	5	24 (39%)	34 (55%)	20 (32%)	25 (40%)	62	5 (28%)	4 (22%)	3 (17%)	8 (44%)	18
2020	27 (42%)	31 (48%)	29 (45%)	28 (44%)	64	23 (39%)	28 (47%)	28 (47%)	24 (41%)	59	4 (80%)	3 (60%)	1 (20%)	4 (80%)	5	23 (46%)	27 (54%)	24 (48%)	23 (46%)	50	4 (29%)	4 (29%)	5 (36%)	5 (36%)	14
2021	31 (39%)	40 (50%)	22 (28%)	27 (34%)	80	28 (37%)	38 (51%)	21 (28%)	25 (33%)	75	3 (60%)	2 (40%)	1 (20%)	2 (40%)	5	25 (36%)	35 (50%)	18 (26%)	22 (31%)	70	6 (60%)	5 (50%)	4 (40%)	5 (50%)	10
2022	25 (30%)	42 (52%)	24 (30%)	31 (39%)	80	21 (28%)	37 (49%)	21 (28%)	27 (36%)	75	4 (80%)	5 (100%)	3 (60%)	4 (80%)	5	23 (32%)	39 (55%)	22 (31%)	28 (39%)	71	2 (22%)	3 (33%)	2 (22%)	3 (33%)	9
2023	33 (41%)	37 (46%)	22 (28%)	33 (41%)	80	29 (39%)	33 (44%)	18 (24%)	29 (39%)	75	4 (80%)	4 (80%)	4 (80%)	4 (80%)	5	30 (42%)	34 (48%)	21 (30%)	28 (39%)	71	3 (33%)	3 (33%)	1 (11%)	5 (56%)	9

The questions with the same questions and choices were as follows: 2023-62 and 2019-66 (single-answer with images).

Supplemental table 2 Accuracy rates of GPT4, GPT4V, and GPT4o-based ChatGPT, Claude3 Sonnet, and Claude3 Opus on the Japanese nuclear medicine board certification test

	All questions					Question with images					Question without images				
	GPT4 and 4V-ChatGPT	GPT4o-ChatGPT	Claude3 Sonnet	Claude3 Opus	No. of all questions	GPT4 and 4V-ChatGPT	GPT4o-ChatGPT	Claude3 Sonnet	Claude3 Opus	No. of question with images	GPT4 and 4V-ChatGPT	GPT4o-ChatGPT	Claude3 Sonnet	Claude3 Opus	No. of question without images
2019	27 (45%)	37 (62%)	16 (27%)	19 (32%)	60	4 (17%)	9 (38%)	6 (25%)	8 (33%)	24	23 (64%)	28 (78%)	10 (28%)	11 (31%)	36
2020	32 (53%)	34 (57%)	13 (22%)	28 (47%)	60	8 (47%)	9 (53%)	2 (12%)	7 (41%)	17	24 (56%)	25 (58%)	11 (26%)	21 (49%)	43
2021	20 (33%)	41 (68%)	16 (27%)	27 (45%)	60	5 (28%)	13 (72%)	4 (22%)	8 (44%)	18	15 (36%)	28 (67%)	12 (29%)	19 (45%)	42
2022	35 (58%)	45 (75%)	21 (35%)	24 (40%)	60	9 (56%)	12 (75%)	9 (56%)	9 (56%)	16	26 (59%)	33 (75%)	12 (27%)	15 (34%)	44
2023	35 (58%)	34 (57%)	17 (28%)	27 (45%)	60	9 (53%)	11 (65%)	6 (35%)	8 (47%)	17	26 (60%)	23 (53%)	11 (26%)	19 (44%)	43

Supplemental table 3 Accuracy rates of GPT4, GPT4V, and GPT4o-based ChatGPT, Claude3 Sonnet, and Claude3 Opus on the Japanese interventional radiology board certification test

	All questions					Question with images					Question without images					Single-answer question					Multi-answer question				
	GPT4 and 4V-ChatGPT	GPT4o-ChatGPT	Claude3 Sonnet	Claude3 Opus	No. of all questions	GPT4 and 4V-ChatGPT	GPT4o-ChatGPT	Claude3 Sonnet	Claude3 Opus	No. of question with images	GPT4 and 4V-ChatGPT	GPT4o-ChatGPT	Claude3 Sonnet	Claude3 Opus	No. of question without images	GPT4 and 4V-ChatGPT	GPT4o-ChatGPT	Claude3 Sonnet	Claude3 Opus	No. of single-answer question	GPT4 and 4V-ChatGPT	GPT4o-ChatGPT	Claude3 Sonnet	Claude3 Opus	No. of multi-answer question
2019	18 (30%)	35 (58%)	22 (37%)	32 (53%)	60	7 (29%)	11 (46%)	6 (25%)	9 (38%)	24	11 (28%)	24 (60%)	16 (40%)	23 (57%)	40	10 (38%)	20 (77%)	13 (50%)	16 (62%)	26	8 (24%)	15 (44%)	9 (26%)	16 (47%)	34
2020	23 (38%)	31 (52%)	19 (32%)	25 (42%)	60	7 (41%)	6 (35%)	2 (12%)	4 (24%)	17	16 (33%)	25 (51%)	17 (35%)	21 (43%)	49	11 (44%)	18 (72%)	7 (28%)	12 (48%)	25	12 (34%)	13 (37%)	12 (34%)	13 (37%)	35
2021	28 (47%)	25 (42%)	19 (32%)	26 (43%)	60	2 (11%)	4 (22%)	3 (17%)	3 (17%)	18	26 (55%)	21 (45%)	16 (34%)	23 (49%)	47	15 (45%)	17 (52%)	11 (33%)	15 (45%)	33	13 (48%)	8 (30%)	8 (30%)	11 (41%)	27
2022	16 (25%)	20 (31%)	13 (20%)	22 (34%)	65	4 (25%)	4 (25%)	7 (44%)	5 (31%)	16	12 (29%)	16 (38%)	6 (14%)	16 (38%)	42	7 (23%)	10 (33%)	8 (27%)	10 (33%)	30	9 (26%)	10 (29%)	5 (14%)	11 (31%)	35
2023	26 (32%)	27 (34%)	25 (31%)	27 (34%)	80	9 (53%)	8 (47%)	9 (53%)	8 (47%)	17	17 (34%)	19 (38%)	16 (32%)	19 (38%)	50	15 (45%)	13 (39%)	13 (39%)	13 (39%)	33	11 (23%)	14 (30%)	12 (26%)	14 (30%)	47

The questions with the same questions and choices were as follows: 2023-6 and 2022-11 (single-answer without images), 2023-62 and 2019-45 (multiple-answer without images), and 2023-75 and 2022-61 (single-answer with images).
